# Glucagon promotes colon cancer cell growth via regulating AMPK and MAPK pathways

**DOI:** 10.18632/oncotarget.24367

**Published:** 2018-01-31

**Authors:** Takashi Yagi, Eiji Kubota, Hiroyuki Koyama, Tomohiro Tanaka, Hiromi Kataoka, Kenro Imaeda, Takashi Joh

**Affiliations:** ^1^ Department of Gastroenterology and Metabolism, Nagoya City University Graduate School of Medical Sciences, Mizuho-ku, Nagoya 467-8601, Japan

**Keywords:** glucagon, colon cancer, proliferation, incretin, glucagon signalling

## Abstract

Cancer is one of the major causes of death in diabetic patients, and an association between antidiabetic drugs and cancer risk has been reported. Such evidence implies a strong connection between diabetes and cancer. Recently, glucagon has been recognized as a pivotal factor implicated in the pathophysiology of diabetes. Glucagon acts through binding to its receptor, glucagon receptor (GCGR), and cross-talk between GCGR-mediated signals and signaling pathways that regulate cancer cell fate has been unveiled. In the current study, expression of GCGR in colon cancer cell lines and colon cancer tissue obtained from patients was demonstrated. Glucagon significantly promoted colon cancer cell growth, and GCGR knockdown with small interfering RNA attenuated the proliferation-promoting effect of glucagon on colon cancer cells. Molecular assays showed that glucagon acted as an activator of cancer cell growth through deactivation of AMPK and activation of MAPK in a GCGR-dependent manner. Moreover, a stable GCGR knockdown mouse colon cancer cell line, CMT93, grew significantly slower than control in a syngeneic mouse model of type 2 diabetes with glycemia and hyperglucagonemia. The present observations provide experimental evidence that hyperglucagonemia in type 2 diabetes promotes colon cancer progression via GCGR-mediated regulation of AMPK and MAPK pathways.

## INTRODUCTION

The number of patients with type 2 diabetes is increasing every year worldwide [1]. Many of these patients have complications related to cardiovascular and cerebrovascular events, eventually leading to death. Previous meta-analyses showed that cancer is the second most common cause of death in diabetes patients [2]. Several studies have suggested a correlation between diabetes and neoplasms, including liver cancer, pancreatic cancer, and colorectal cancer [3, 4]. Furthermore, diabetes medications including metformin and α-glucosidase inhibitors have been reported to reduce the risk of cancer incidence [5–7]. In contrast, use of insulin has been shown to be associated with an increased risk of cancer in type 2 diabetes patients [8, 9]. In addition to the etiological correlation between diabetes and cancer, experimental studies have proven the mechanisms responsible for cancer predisposition in diabetes, including hyperglycemia, insulin resistance, and chronic inflammation [10, 11], and the mechanisms how antidiabetic agents inhibit cancer growth [12–14]. This accumulated evidence suggests that diabetes is implicated in cancer development and progression.

Interestingly, recent studies have demonstrated that blood glucagon levels are increased in diabetes patients, and hyperglucagonemia plays a crucial role in the pathogenesis of diabetes [15–17]. Glucagon, a 29-amino acid peptide, is secreted from the α-cells in the pancreas, and part of it is also secreted from the endocrine cells in the gastrointestinal tract [18]. Glucagon works through binding to its receptor, a glucagon receptor (GCGR), which is expressed in various organs, including liver, kidney, brain, intestinal tract, pancreas, and adipose tissue [19]. A main physiological function of glucagon is increasing the blood glucose level by promoting hepatic glucose output through binding to GCGRs on hepatocytes [20]. Glucagon has many functionalities, including increased lipolysis in adipose tissue, action as a satiety factor in the CNS, regulatory effects on glomerular filtration, reduction of gastrointestinal motility, and inducing proliferation of mesangial cells [19]. As for the role of glucagon in cancer, glucagon has been shown to enhance growth of cultured human colorectal cancer cells *in vitro* [21]. However, the mechanism of how glucagon promotes colon cancer cell proliferation has not yet been determined.

Taken together with this background on glucagon, we hypothesized that hyperglucagonemia is one factor that increases the risk of colorectal cancer in diabetes patients. In this study, the direct effect of glucagon on colon cancer was investigated, and the glucagon mechanism of action as an activator of colon cancer cell proliferation *in vitro* was elucidated. Moreover, it was demonstrated that hyperglucagonemia promotes colon cancer cell growth *in vivo* using a syngeneic mouse cancer model with type 2 diabetes.

## RESULTS

### GCGR is expressed in human colon cancer cell tissue and colon cancer cell lines

**Figure 1:**
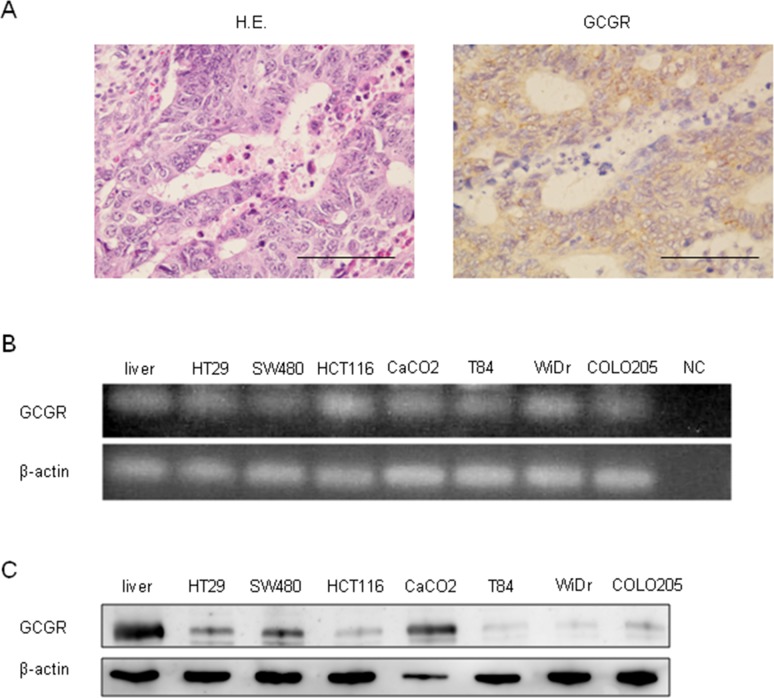
GCGR expression Expression of GCGR in human colon cancer tissue and colon cancer cell lines **(A)** Representative images of hematoxylin and eosin (HE) and GCGR staining of human colon cancer tissue obtained from patients with colon cancer. Scale bar, 100μm. **(B)** Expressions of GCGR mRNA in seven human colon cancer cell lines evaluated by RT-PCR. Normal human liver mRNA is used as a positive control. cDNA replaced with water is used as negative control (NC). **(C)** Expressions of GCGR protein in seven human colon cancer cell lines evaluated by western blot analysis. Normal human liver protein is used as a positive control.

The expression of GCGR was evaluated in human colon cancer tissues obtained from patients. Immunohistochemical staining showed that GCGR was expressed in colon cancer tissue (Figure 1A). Real-time PCR and western blot analysis demonstrated that GCGR was expressed in seven human cancer cell lines including HT29, SW480, HCT116, CaCO2, T84, WiDr, and COLO205 (Figure 1B, 1C).

### Glucagon promotes human colon cancer proliferation via activation of GCGR

**Figure 2:**
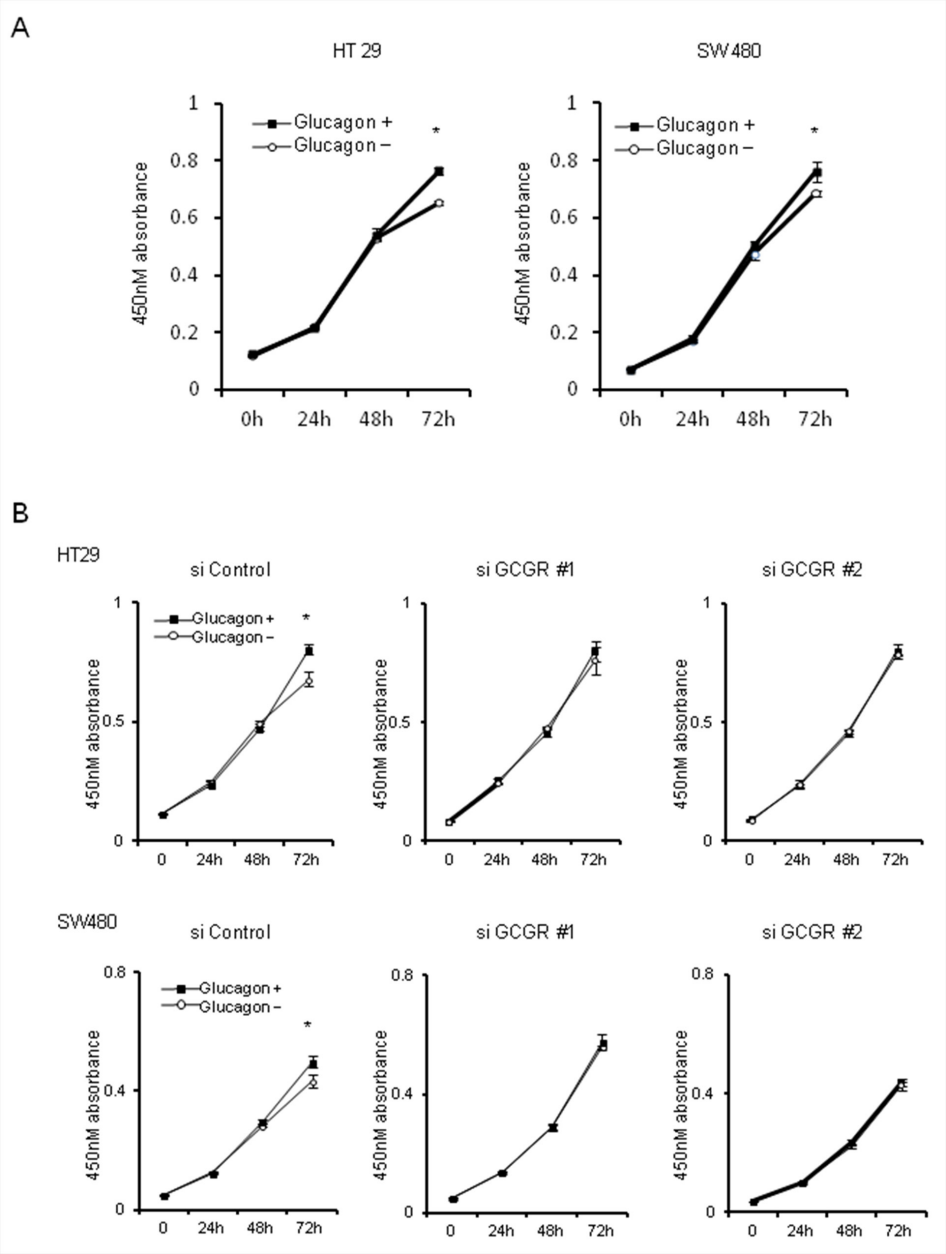
Tumor growth with glucagon Glucagon promotes proliferation of human colon cancer cell lines via GCGR activation **(A)** HT29 and SW480 cells were incubated in recommended culture media with or without stimulation by 1.0 nM of glucagon every 24 h. After 24, 48, and 72 h, cell viability was measured using an MTT assay. **(B)** HT29 and SW480 cells transfected with siRNA for control or GCGR were incubated in recommended culture media with or without stimulation by 1.0 nM glucagon every 24 h. After 24, 48, and 72 h, cell viability was measured using an MTT assay. Data are presented as means ± SD, *n*=3. A significant difference is indicated as ^*^ (p < 0.05, Student's *t*-test).

The effect of glucagon on cell proliferation was investigated using the MTT assay. Glucagon stimulation was performed by supplying 1.0 nM glucagon onto culture media every 24 h to maintain the concentration of glucagon, because glucagon was not stable in culture media (Supplementary Figure 1). Glucagon significantly promoted the proliferation of HT29 and SW480 at 72 h after incubation with culture medium supplemented with 1.0 nM of glucagon every 24 h (Figure 2A). To investigate whether glucagon enhances cell growth through activation of GCGR, knockdown of GCGR in HT29 and SW480 was performed by siRNA, and GCGR knockdown cells were then incubated with or without glucagon. Western blotting showed that an approximately 70% reduction of GCGR expression was induced by siRNA in both HT29 and SW480 (Supplementary Figure 2). As shown in Figure 2B, suppression of GCGR by siRNA abolished the promoting effect of glucagon on cell proliferation in both H29 and SW480 at 72 h after incubation with glucagon. These results indicated that glucagon directly stimulated colon cancer cell growth through binding to GCGR.

### Molecular responses after glucagon stimulation in human colon cancer cells

**Figure 3:**
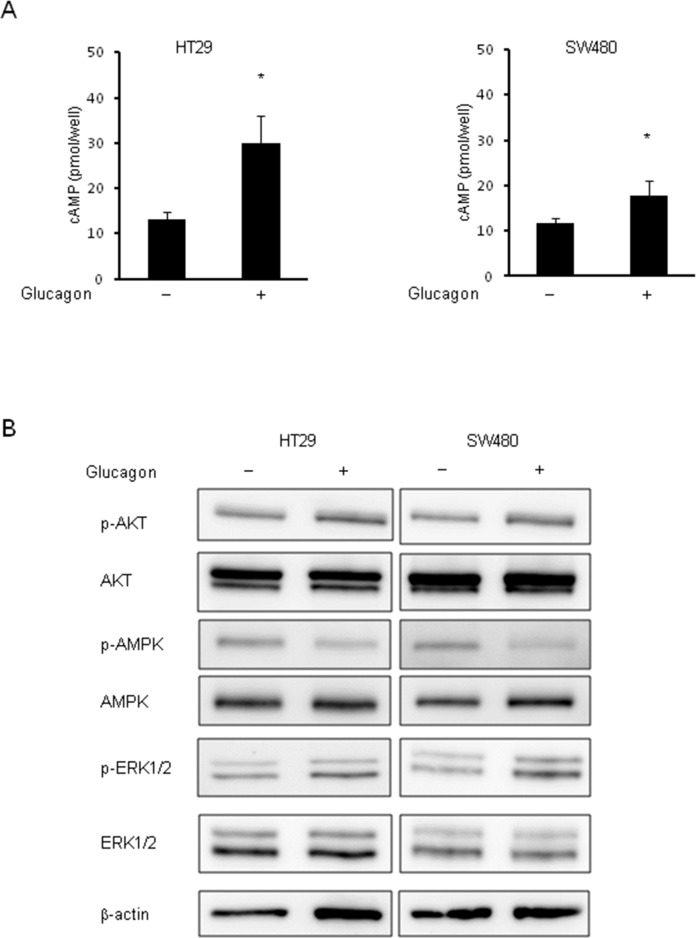
Phosphorylation of AMPK and ERK by glucagon Glucagon reduces the activity of phosphorylation of AMPK and induces phosphorylation of ERK1/2 in human colon cancer cell lines. **(A)** Concentrations of intracellular cAMP were measured in HT29 and SW480 treated with or without 1.0 nM glucagon for 15 min. **(B)** Protein extract from HT29 and SW480 cells incubated in culture media supplemented with or without 1.0 nM glucagon for 1 h was immunoblotted with anti-phospho-AKT (Ser473), anti-AKT, anti-phospho-AMPKα (Tyr172), anti-AMPKα, anti-phospho-ERK1/2 (Thr202/Tyr204), and anti-ERK1/2 antibodies. β-actin is shown as a loading control. Data are presented as means ± SD, *n*=5. A significant difference is indicated as ^*^ (p < 0.05, Student's *t*-test).

To determine whether glucagon is functional in GCGR expressing colon cancer cell lines, we measured intracellular cAMP concentrations in HT29 and SW480 with or without glucagon stimulation. In response to glucagon, the concentrations of intracellular cAMP were significantly increased in both HT29 and SW480 (Figure 3A). Furthermore, to identify the mechanisms promoting colon cancer cell growth by glucagon, the activation of downstream signals of GCGR was examined by western blotting using colon cancer cells treated with glucagon. AKT, AMPK, and MAPK pathways are intracellular signals that are involved in colon cancer progression [22–24]. These pathways are also regulated through GCGR signaling mediated by glucagon [25–28]. The phosphorylation of AMPK was inhibited by glucagon stimulation in HT29 and SW480. In contrast, glucagon increased the phosphorylation level of ERK1/2 compared to control. There was no significant difference in AKT phosphorylation between control and cells treated with glucagon (Figure 3B). Taken together, these results showed that glucagon enhances colon cancer cell growth through regulation of proliferative signaling including the AMPK and MAPK pathways.

### Glucagon promotes mouse colon cancer cell proliferation via activation of GCGR

**Figure 4:**
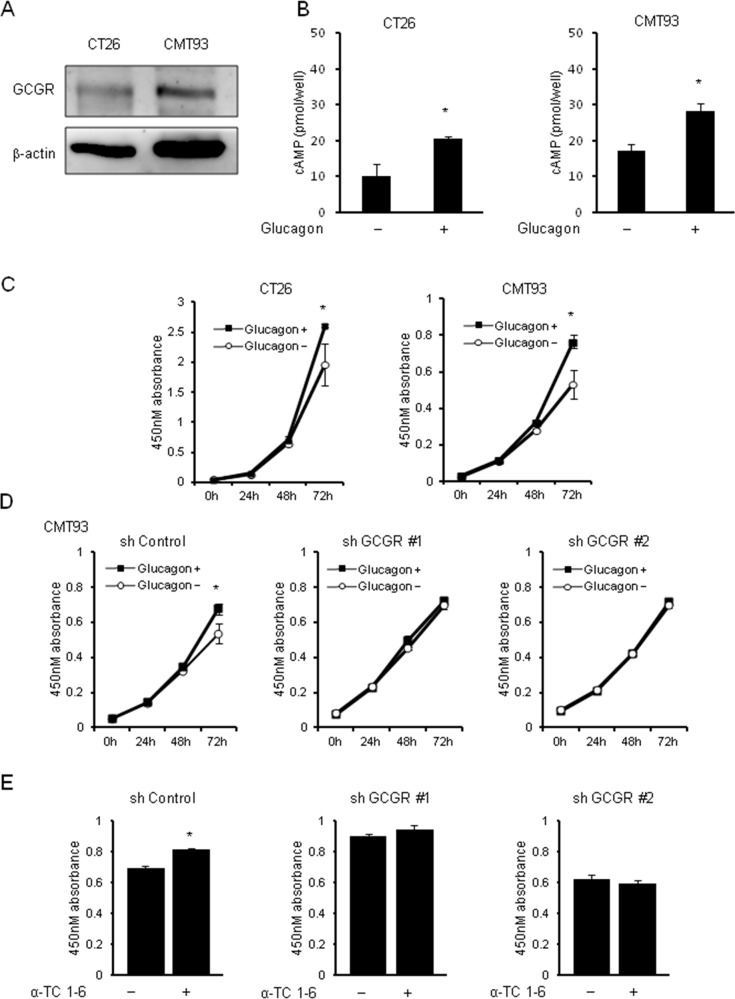
Tumor growth with glucagon in mouse colon cancer cell lines Glucagon promotes the proliferation of mouse colon cancer cells via activation of GCGR expressed in mouse colon cancer cells. **(A)** Expressions of GCGR protein in mouse colon cancer cell lines CT26 and CMT93 evaluated by western blot analysis. **(B)** Concentrations of intracellular cAMP were measured in CT26 and CMT93 treated with or without 1.0 nM glucagon for 15 min. Data are presented as means ± SD, *n*=5. **(C)** CT26 and CMT93 cells were incubated in recommended culture media with or without stimulation by 1.0 nM glucagon every 24 h. After 24, 48, and 72 h, cell viability was measured using an MTT assay. **(D)** CMT93 cells transfected with shRNA for control or GCGR were incubated in recommended culture media with or without stimulation by 1.0 nM glucagon every 24 h. After 24, 48, and 72 h, cell viability was measured using an MTT assay. Data are presented as means ± SD, *n*=3. **(E)** CMT93 cells transfected with shRNA for control or GCGR were co-incubated with or without α-TC1-6 cell, cell viability was measured using an MTT assay. Data are presented as means ± SD, *n*=3. A significant difference is indicated as ^*^ (p < 0.05, Student's *t*-test).

To investigate the effect of hyperglucagonemia on colon cancer progression using the type 2 diabetes mouse model, mouse colon cancer cell lines, CT26 and CMT93, which are extensively used as a syngeneic tumor model, were used. First, GCGR expression was investigated in CT26 and CMT93. Western blotting showed the expressions of GCGR in CT26 and CMT93 (Figure 4A). To ascertain the functionality of glucagon in mouse colon cancer cell lines, we measured changes in intracellular cAMP concentrations before and after glucagon stimulation. Glucagon induced significant increase of intracellular cAMP concentrations in CT26 and CMT93 (Figure 4B). Cell proliferation assays were performed and demonstrated that cell growth was significantly increased in both CT26 and CMT93 cells cultured with 1.0 nM of glucagon for 72 h as compared to cells without glucagon treatment (Figure 4C). Additionally, we found that glucagon increased the ratio of Bromodeoxyuridine (BrdU)-positive CMT93 cells (Supplementary Figure 3A). Next, GCGR knockdown CMT93 clones were established using shRNA to GCGR (shGCGR) expression vector. The expressions of GCGR mRNA and GCGR protein were significantly suppressed in GCGR knockdown clones compared to control (Supplementary Figure 4A and 4B). A cell viability assay demonstrated that stimulation of glucagon enhanced the proliferation of CMT93 transfected with scramble shRNA, whereas glucagon did not show any effect on growth of GCGR knockdown clones (Figure 4D). Furthermore, the proliferation of CMT93 co-incubated with mouse pancreatic alpha-cells, α-TC1-6, which secrete glucagon, was evaluated using a trans-well system. The concentration of glucagon in culture medium of CMT93 co-cultured with α-TC1-6 was stable as compared to culture medium supplemented with 1.0 nM of glucagon every 24 h (Supplementary Figure 5). CMT93 cells co-cultured with α-TC1-6 showed a significant increase in cell proliferation. In contrast, no promoting effects were seen in the proliferation of GCGR knockdown clones co-cultured with α-TC1-6 (Figure 4E). Without glucagon stimulation, GCGR knockdown did not show any change in cell growth (Supplementary Figure 4C). These results are in accordance with the results of experiments using human colon cancer cell lines.

### Glucagon modulates phosphorylation of AMPK and MAPK pathways via GCGRs in mouse colon cancer cells

**Figure 5:**
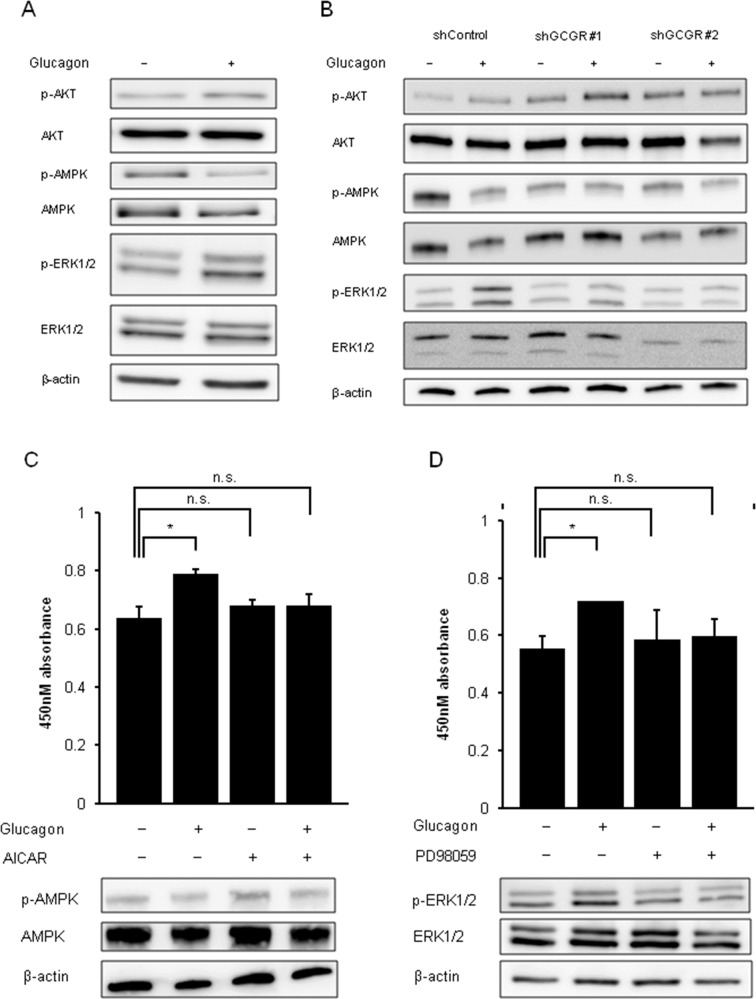
Phosphorylation of AMPK and phosphorylation of ERK by glucagon Glucagon promotes mouse colon cancer cell growth via regulating AMPK and MAPK pathways in a GCGR-dependent manner. **(A)** Protein extracted from CMT93 cells incubated in culture media supplemented with or without 1.0 nM glucagon for 30 min was immunoblotted with anti-phospho-AKT (Ser473), anti-AKT, anti-phospho-AMPKα (Tyr172), anti-AMPKα, anti-phospho-ERK1/2 (Thr202/Tyr204), and anti-ERK1/2 antibodies. β-actin is shown as a loading control. **(B)** Protein extracted from control and GCGR knockdown CMT93 clones incubated in culture media supplemented with or without 1.0 nM glucagon for 30 min were immunoblotted with anti-phospho-AKT (Ser473), anti-AKT, anti-phospho-AMPKa (Tyr172), anti-AMPKa, anti-phospho-ERK1/2 (Thr202/Tyr204), and anti-ERK1/2 antibodies. β-actin is shown as a loading control. **(C)** CMT93 cells were treated with or without glucagon in combination with or without 250 μM of AICAR for 72 h for cell viability assay and western blot analysis. **(D)** CMT93 cells were treated with or without glucagon in combination with or without 500 mM PD98059 for 72 h for the cell viability assay and western blot analysis. β-actin is shown as a loading control. Data are presented as means ± SD, *n*=3. A significant difference is indicated as ^*^ (p < 0.05, Student's *t*-test).

The effect of glucagon on GCGR-mediated signaling pathways was investigated using mouse colon cancer cell lines CMT93 and CT26. Glucagon stimulation reduced the phosphorylation of AMPK, whereas it enhanced the phosphorylation of ERK1/2 in CMT93 (Figure 5A). We did not detect significant changes in phosphorylation level of AKT. Glucagon similarly regulated these signals in CT26 (Supplementary Figure 6). To further verify whether glucagon modulates intracellular signaling pathways via GCGRs for affecting CMT93 cell proliferation, the phosphorylation of AKT, AMPK, and ERK1/2 was investigated in GCGR knockdown CMT93. As shown in Figure 5B, glucagon stimulation significantly reduced the phosphorylation of AMPK in control, whereas significant changes in the phosphorylation level of AMPK after glucagon treatment were not seen in GCGR knockdown clones. Although phospho-ERK1/2 levels were significantly increased in control treated with glucagon, glucagon did not enhance the activity of ERK1/2 in GCGR knockdown clones.

The effect of the activator of AMPK, AICAR, or the specific inhibitor of MAPK, PD98059, on cell proliferation of CMT93 stimulated by glucagon was examined to investigate whether these factors are involved in glucagon-mediated promotion of colon cancer cell growth. The maximum concentrations of AICAR or PD98059 that hinder the regulatory effect of glucagon on signal transduction without cell toxicity in CMT93 were determined (data not shown here). Combination treatment with AICAR and glucagon inhibited the deactivation of AMPK induced by glucagon in CMT93. Moreover, AICAR partially attenuated the promoting effect of glucagon on proliferation of CMT93 (Figure 5C). PD98059 inhibited ERK1/2 activation stimulated by glucagon and induced a partial reduction in the promoting effect of glucagon on cell growth in CMT93 (Figure 5D). These observations indicate that glucagon promotes cell proliferation of colon cancer cells through regulation of downstream mediators of GCGR involving AMPK, and MAPK.

### Effect of glucagon on growth of mouse colon cancer cells in type 2 diabetes model mice

**Figure 6:**
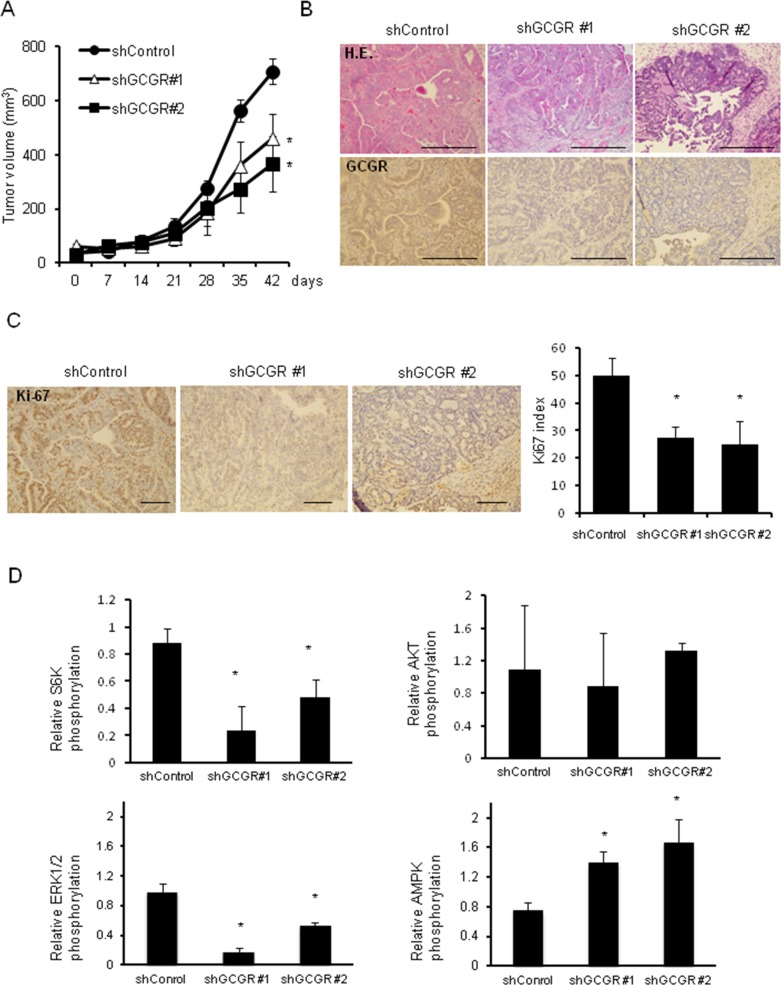
Tumor growth in type 2 diabetes model mice GCGR knockdown significantly inhibits the growth of allograft tumors in type 2 diabetes model mice **(A)** 5.0×10^6^ CMT93, control cells, and GCGR knockdown clones were subcutaneously injected into C57BL/6-DOI mice. Tumor growth was measured once a week. Data are presented as means ± SE, 5 animals per group. A significant difference is indicated as ^*^ (p < 0.05, ANOVA). **(B)** Representative images of hematoxylin and eosin (HE) and GCGR staining of subcutaneously transplanted control and GCGR knockdown clones in C57BL/6-DOI mice. Scale bar, 100μm. **(C)** Staining and enumeration of Ki-67-positive cells in allografts of control and GCGR knockdown clones. Data are presented as means ± SD. A significant difference is indicated as ^*^ (p < 0.05, ANOVA). Scale bar, 100μm. **(D)** Relative phosphorylation of S6K, AKT, AMPK and ERK1/2 proteins quantified by densitometry using imageJ software. Data are presented as means ± SD, *n*=3. A significant difference is indicated as ^*^ (p < 0.05, Student's *t*-test).

Whether glucagon stimulates tumor growth of CMT93 was examined using type 2 diabetes model mouse allografts. Body weights, absolute plasma glucose levels, and plasma glucagon levels in type 2 diabetes model mice, C57BL/6-DIO mice, are shown in Table 1. Body weights of C57BL/6-DIO mice were approximately 1.5-fold greater than of control mice. Plasma glucagon levels in C57BL/6-DIO mice were around 2 times higher than in control mice at all measured points. Hyperglycemia was observed in C57BL/6-DIO mice. Next, the differences in tumor growth between GCGR knockdown CMT93 clones and control allografts in C57BL/6 and C57BL/6-DIO mice were evaluated. Tumor in C57BL/6-DIO mice transplanted GCGR knockdown clones grew significantly slower than in mice transplanted control (Figure 6A, Supplementary Figure 9). In contrast, there were no significant differences in tumor growth between GCGR knockdown clones and control in C57BL/6 mice (Supplementary Figure 7). Moreover, tumors transplanted into C57BL/6 mice grew slower than tumors in C57BL/6-DIO mice irrespective of GCGR expression status. Histopathological analyses of extracted tumors from mice revealed that tumor cells are prone to be surrounded by stromal tissue in transplanted tumor of GCGR knockdown clones (Figure 6B). Though GCGR was diffusely expressed in control, GCGR expression was markedly decreased in GCGR knockdown clone allografts (Figure 6B). The numbers of Ki67-positive cells in CGCR knockdown clone allografts were significantly decreased compared to in control (Figure 6C). Western blot analysis of extracted protein from mice allografts showed that GCGR knockdown affected the phosphorylation levels of AKT, AMPK, ERK1/2 and S6K (Supplementary Figure 8). Although there were variable expression patterns, GCGR knockdown showed significant elevation of AMPK phosphorylation compared to control when averaged over 3 tumors. In contrast, GCGR knockdown significantly reduced phosphorylation of S6K and ERK1/2 relative to control, which had higher levels of phosphorylated proteins (Figure 6D). The findings in type 2 diabetes model mouse allografts are in line with the results of *in vitro* studies using human colon cancer cell lines.

**Table 1:** Characteristics of C57BL/6-DIO mice

Characteristics	C57BL/6		C57BL/6-DIO	
Week-old	10	17	10	17
Body weight (g)	27.3 ± 0.97	28.8 ± 1.6	32.4 ± 0.9	37.4 ± 2.8
Plasma blood glucose (mg/dl)	279.0 ± 92.1	280.5 ± 74.9	399.3 ± 74.4	418.4 ± 137.7
Plasma blood glucagon (pM)	6.8 ± 2.5	5.7 ± 1.8	13.2 ± 4.9	14.3 ± 4.5

## DISCUSSION

Since GCGR was identified by Jelinek et al. [29], many studies have shown the glucagon-mediated signaling effects through glucagon binding to GCGR, a G protein-coupled receptor. Glucagon works mainly to increase blood glucose levels through activating glycogenolytic and gluconeogenic pathways, and recent evidence suggests that glucagon is a key contributing factor to the development and progression of diabetes [19, 30]. Accumulating evidence about glucagon has promoted the development of novel drugs regulating glucagon in patients with diabetes, and clinical trials of these agents for diabetes are underway [19]. Beyond glucose homeostasis, glucagon is known to show a wide range of biological activities. However, the pathophysiological role of glucagon in cancer is not well known. Although a previous study demonstrated the stimulating effect of glucagon on the growth of human colorectal adenocarcinoma cells obtained from patients *in vitro*, the mechanism of how glucagon stimulates colon cancer cell proliferation remains unclear [21].

In this study, GCGR mRNA and protein expression were demonstrated in colon cancer cell lines by RT-PCR and western blotting respectively, and immunohistochemical staining showed GCGR expression in colon cancer tissue. We observed that glucagon induced significant increase in the concentrations of intracellular cAMP, a second messenger that allows GCGR to activate downstream molecular targets.

Besides that, glucagon was found to enhance colon cancer cell proliferation, whereas glucagon did not promote cell proliferation in GCGR knockdown colon cancer cells. These results indicate that glucagon elicits direct biological actions through the GCGR, which is expressed in colon cancer cells. Additionally, GCGR-mediated signals involving colon cancer cell growth were investigated to elucidate the mechanism of how glucagon regulates cancer cell growth. First, the focus was on the activation of MAPK signaling mediated by glucagon, which governs the development and progression of colorectal cancer [23, 31]. Previous reports suggested that glucagon acts as an active moderator of ERK1/2. Glucagon stimulates ghrelin secretion in a dose-dependent manner through activating the MAPK pathway in rat stomach culture cells [32]. Another study reported that glucagon-induced ERK1/2 phosphorylation contributes positively to mesangial cell proliferation [26]. In the current study, glucagon was found to increase phosphorylation of ERK1/2 in colon cancer cells, and glucagon did not phosphorylate ERK1/2 in GCGR knockdown colon cancer cells, resulting in attenuation of the promoting effect of glucagon on cell growth. In addition, PD98059, an inhibitor of MAPK, cancelled the promoting effect of glucagon on cell proliferation, though PD98059 alone did not show any effect on cell viability. These results indicate that glucagon directly promotes colon cancer cell growth by activation of MAPK through binding to GCGR. Glucagon-like-peptide-1 (GLP-1) agonist, which also binds to G-protein coupled receptors and works as a stimulator of cAMP production [33, 34], promotes mouse osteoblastic cells and cardiac microvascular endothelial cells via the activation of MAPK signaling pathways [35, 36]. Meanwhile, GLP-1 agonist inhibits prostate cancer growth through inhibition of ERK despite of its promotive effect on cAMP production [14]. The activation of ERK is likely to be regulated by cAMP in a cell-specific manner. Although the mechanism involved in the inhibition or activation of ERK signaling by cAMP is in part explained by genetic status of cells, it remains to be clarified completely [37]. In this study, we did not detect the effect of glucagon on AKT activation *in vitro* and *in vivo* studies.

Next, AMPK signaling regulated by glucagon in colon cancer cells was investigated. AMPK plays a key role as a master regulator of cellular energy homeostasis by phosphorylation of enzymes involved in metabolic pathways [38]. There is also emerging evidence for a role of AMPK in the regulation of cancer cell growth and survival [38–40]. Therefore, AMPK is recognized as a therapeutic target not only for type 2 diabetes, but also for cancer. Indeed, metformin, an AMPK activator, which is widely used to treat diabetes patients, has been reported to reduce the risk of cancer in diabetes patients [6, 7]. Other reports suggest that activation of AMPK by metformin inhibits cancer cell proliferation [41, 42]. Recently, metformin has been shown to antagonize the glucagon effect through AMPK, which results in reduction of glucose levels [43, 44]. In the present study, glucagon suppressed the phosphorylation of AMPK at Thr172 in colon cancer cells through activation of the GCGR pathway. The present results are not in line with the previous report, which demonstrated glucagon-induced phosphorylation of AMPK at Thr172 in rat liver [28]. Darius et al. demonstrated that glucagon decreased activating phosphorylation of AMPK at Thr172 in a human liver cell line, which suggested that glucagon works as an AMPK regulator in a species-specific manner [25]. In addition to the species-specific effect of glucagon, differences in origin of the cell or pathophysiological background where glucagon acts as an AMPK manipulator may account for this discrepancy. Although further studies are needed to identify the detailed mechanisms of how glucagon regulates AMPK, we believe that glucagon induces inhibitory activity against AMPK and consequently enhances cell growth in colon cancer cells.

In the present study, the *in vitro* experiments showed a modest effect of glucagon on colon cancer cell proliferation compared to *in vivo* studies. This may be due to the instability and short half-life of glucagon protein. Glucagon is an unstable protein that immediately undergoes degradation and aggregation in aqueous solution. Indeed, the concentration of glucagon in culture medium decreased immediately (Supplementary Figure 1). Additionally, *in vitro* studies showed the short-term effect of glucagon on cancer cells, which could not clearly elucidate the role of hyperglucagonemia on cancer cell progression. In type 2 diabetes model mice, there were distinct differences in tumor growth between control and GCGR knockdown clones, even though the concentrations of plasma blood glucagon in mice are much lower than in cell culture media used for *in vitro* studies. There was also a significant decrease in the number of Ki-67-positive cells in tumors of GCGR knockdown clones (Figure 6C). Furthermore, GCGR knockdown abolished the glucagon-mediated deactivation of AMPK and activation of MAPK (Figure 6D), resulting in induction of an inhibitory effect of tumor growth in type 2 diabetes model mice. In mice fed with normal diet whose concentration of glucagon was approximately one-half of that in type 2 diabetes model mice (Table 1), GCGR knockdown did not show a significant effect on tumor growth (Supplementary Figure 7). These results indicate that hyperglucagonemia might be one of the mechanisms responsible for cancer progression in type 2 diabetes patients.

In conclusion, GCGR expression was detected in colon cancer tissues obtained from patients. Moreover, glucagon was demonstrated to promote colon cancer cell proliferation through binding to GCGR expressed in human and mouse colon cancer cell lines. It was also shown that downstream signals of GCGR, including AMPK and MAPK pathways, play a crucial role in colon cancer proliferation *in vitro* and *in vivo*. These observations demonstrate the significance of hyperglucagonemia in colon cancer progression and suggest that glucagon activities might be potential therapeutic targets for colon cancer patients with type 2 diabetes.

## MATERIALS AND METHODS

### Reagents

PD98059, a specific inhibitor of the mitogen-activated protein kinase (MAPK)/extracellular signal-regulated kinase (ERK), 5-aminoimidazole-4-carboxamide 1-β-D-ribofuranoside (AICAR), an activator of AMP-activated protein kinase (AMPK), and glucagon were obtained from Sigma-Aldrich (St. Louis, MO, USA).

### Cell culture

The human colon cancer cell lines, HT29, SW480, HCT116, CaCo2, WiDr, T84, COLO205, mouse colon cancer cell lines, CMT93, CT26, and the murine pancreatic α-cell line, α-TC1-6 cells (American Type Culture Collection (ATCC), Manassas, VA, USA) were used in this study. HT29 and HCT116 were cultured in McCoy's 5A (Thermo Fisher Scientific K.K, Kanagawa, Japan), SW480 was cultured in an equal mixture of Dulbecco's modified Eagle's medium (DMEM) and Ham's F-12 (Wako Pure Chemical Industries Co. Ltd., Osaka, Japan), WiDr was cultured in Eagle's minimal essential medium (EMEM; Wako), COLO205 was cultured in RPMI-1640 (Wako), CMT93 and CT26 were cultured in DMEM, alpha-TC1-6 (α-TC1-6) was cultured in DMEM, 15 mM HEPES, 0.1 mM non-essential amino acids (Gibco, Thermo Fisher Scientific Corp., Carlsbad, CA, USA), 0.02% bovine serum albumin (Wako), 1.5 g/L sodium bicarbonate (Gibco), 2.0 g/L glucose (Wako), supplemented with 10% fetal bovine serum (FBS) in a 5% CO_2_ atmosphere until examined. T84 was cultured in an equal mixture of DMEM and Ham's F-12, supplemented with 5% FBS, CaCo2 was cultured in EMEM, with 20% FBS in a 5% CO_2_ atmosphere until examined.

### Co-culture of colon cancer cells and pancreatic α-cells

Co-culture of mouse colon cancer cell line CMT93 and mouse pancreatic α-cell line α-TC1-6 was performed using a trans-well system. CMT93 sh Control or CMT93 sh GCGR clones were seeded onto 24-well plates with 3.0×10^3^ cells per well. α-TC1-6 cells were seeded onto 0.4-μm polyester membrane trans-wells (Corning, Glendale, AZ, USA) with 1.0×10^4^ cells, and the trans-wells placed in the 24-well plates seeded with CMT93 shControl or CMT93 shGCGR clones were incubated for cell proliferation assays.

### Cell proliferation analysis

Cells were seeded onto 96-well plates with 1.0×10^3^ cells per well and cultured overnight. After stimulating the cultured cells with chemical agents, cell proliferation was measured using the MTT assay (Cell Counting Kit-8 (CCK-8); Dojindo Laboratories, Kumamoto, Japan) in each plate according to the manufacturer's protocol. Cell culture medium was aspirated and replaced with 10 μl of CCK-8 reagent in 100 μl of culture medium after 1.5 h of incubation at 37°C under 5% CO_2_. The absorbance of each sample was determined using a microplate reader at 450 nM (SPECTRA MAX340; Molecular Devices, Kenilworth, NJ, USA).

### Real–time PCR (RT-PCR)

Total mRNA from colon cancer cells was extracted using simply RNA Cells and Tissue Kit (Promega Corporation, Madison, WI, USA), cDNA was synthesized using cDNA Reverse Transcription Kit (Applied Biosystems, Thermo Fisher Scientific Corp.). Normal human liver mRNA (BioChain Institute Inc., Newark, CA, USA) was used as a positive control for GCGR expression. RT-PCR was performed using Power SYBR green (Thermo Fisher Scientific). Cycling conditions were one cycle at 95 for 2 min and 45 cycles at 95 for 15 sec, 60 for 1 min. Human GCGR primers described previously [45], forward 5′-CGCTGACCCTCATCCCTCCTG-3′ and reverse 5′-TAGAGGACAGCCACCAGCAG-3′, human actin forward 5′-ACAGAGCCTCGCCTTTGC-3′ and reverse 5′-GCGCGGCGATATCATCATCC-3′. The primers sequences of mouse GCGR and 34B4 primers were described previously [46]. PCR products were separated electrophoresis in agarose gel and stained by ethydium bromide.

### Western blot analysis

Cells were plated onto 24-well plates at 1.0×10^5^ cells per well and allowed to grow overnight at 37°C under 5% CO_2_. Cells were stimulated with agents for the appropriate times at 37°C and subsequently scraped on ice in Cytobuster cell lysis buffer (Cell Signaling Technology, Lake Placid, NY, USA) supplemented with protease and phosphatase inhibitor cocktails (Cell Signaling Technology), and then added to sample buffer. Frozen tumors excised from mice were also treated with cell lysis buffer and added to sample buffer. Normal human liver protein sample was obtained from BioChain Institute Inc.. Samples were heated to 100°C for 5 min and stored at −80°C until analysis. Equal amounts of protein were separated by sodium dodecyl sulfate (SDS)-poly-acrylamide gel electrophoresis (PAGE), using a 10% poly-acrylamide gel and transferred onto a nitrocellulose membrane. The membrane was blocked with 5% skim milk in PBS for 1 hour at room temperature, followed by incubation with the primary antibodies against GCGR (Santa Cruz Biotechnology, Dallas, TX), phospho-p70S6K (Thr389), p70S6K, phospho-AMPKα (Tyr172), AMPKα, phosphor-AKT (Ser473), AKT, phospho-ERK1/2 (Thr202/Tyr204), ERK1/2, Cleaved caspase-3 (Asp175), Caspase-3 (Cell Signaling Technology), and β-actin (Abcam, Cambridge, MA, UK) overnight at 4°C. Membranes were fixed in 0.5% milk for 1 h and incubated overnight at 4°C with the respective primary antibodies. Membranes were washed 3 times in 1% phosphate buffered saline Triton X-100 (PBST) and subsequently incubated with species-appropriate secondary antibodies for 1 h. Chemiluminescence was measured using an analyzer, GE Imagequant LAS-4000mini (GE Healthcare, Chicago, IL, USA). Densitometry analysis was performed using ImageJ software [47].

### Measurement of intracellular cAMP

Cells were grown in 10 cm dishes to 70% confluence and were serum starved over night. Cells were detached and plated 2,500 cells per wells in 384-well microplates and stimulated with glucagon for 15 min. Intracellular cAMP concentrations were measured using AlphaScreen cAMP kit (PerkinElmer, Inc. Waltham, MA, USA) according to the manufacturer's instructions.

### Knockdown of GCGR by small interfering RNA (siRNA)

GCGR Stealth siRNA ((Invitrogen, Carlsbad, CA, USA) or negative control siRNA (Invitrogen) was mixed with Lipofectamine RNAiMAX (Invitrogen) in an OptiMEM serum-free medium (Invitrogen) for 5 min at room temperature and then added to cells at a final concentration of 10 pmol/L. Forty-eight hours post transfection, cells were harvested for western blotting and cell proliferation assays.

### Transfection of GCGR-short hairpin RNA (shRNA)

Stable transfection of CMT93 cells was performed with shERWOOD UltramiR shRNA encoding short hairpin RNA (shRNA) to either scrambled or GCGR (Transomic Technologies, Huntsville, AL, USA). Cells were transfected using OMNIfect transfection reagent (Transomic Technologies) according to the manufacturer's instructions. The transfected cells were selected by growth in medium containing 1.5 mg/ml neomycin (Wako, Tokyo, Japan), and single-cell populations were isolated.

### Animal experiments

Eight-week-old male C57BL/6 and C57BL/6-diet induced obesity (C57BL/6-DIO) mice were purchased from Charles River Laboratories Japan, Inc. (Yokohama, Japan), and C57BL/6-DIO were fed a high-fat diet (D12492; Research Diets, Inc., New Brunswick, NJ, USA). The mice were acclimatized for 2 weeks before the experimental procedures. The mice were kept in each individual cage under a 12-h light/dark cycle with free access to water and food. At the age of 10 weeks, CMT93 shControl or shGCGR clones (5.0×10^6^) were subcutaneously injected, and blood samples were collected. The maximum tumor diameter (L) and the diameter at right angles to that axis (W) were measured weekly using calipers, and the volume was calculated according to the formula (L × W^2^)/2. At the age of 17 weeks, blood samples were collected before euthanasia. After euthanasia, transplanted tumors were excised and fixed in formalin or frozen in liquid nitrogen for western blotting and histological analyses. Serum glucagon levels were analyzed by immunoassay using an ELISA kit, which targeted each end of the glucagon peptide (Mercodia AB, Sylveniusgatan Uppsala, Sweden) according to the manufacturer's instructions. Blood glucose levels were measured using One-TouchVerio Vue (Johnson & Johnson, New Brunswick, NJ, USA). The weights of the mice were measured weekly through the experiment. The procedures in these experiments were approved by Nagoya City University Center for Experimental Animal Science, and the mice were cared for according to the guidelines of the Nagoya City University for Animal Experiments.

### Immunohistochemical staining

Deparaffinized samples of human and mouse colon cancer tissues were stained with hematoxylin or incubated with anti-GCGR antibody (1:100, Novas, Littleton, CO, USA) or anti-Ki67 antibody (1:200, Abcam). They were subsequently incubated with biotinylated secondary antibody and then with avidin-biotinylated horseradish peroxidase complex (Histofine, SAB-PO kit, Nichirei Bioscience, Tokyo, Japan). Finally, immune complexes were visualized by incubation with 0.01% H_2_O_2_ and 0.05% 3,3′-diaminobenzidine tetrachloride (DAB). Human colon cancer tissues surgically resected from patients with stage II to III disease at the Department of Gastroenterological Surgery, Nagoya City University Hospital were used. The samples were fixed in formalin embedded in paraffin and cut into 3-μm-thick sections. This study was approved by the institutional review board at Nagoya City University Hospital and each patient provided written, informed consent in this study.

### Data analysis

Data are presented as means and standard deviation or means and standard error of the mean. Significance was determined by Student's *t*-test or the Bonferroni post hoc test. A P value < 0.05 was considered significant.

### DNA synthesis and cell cycle analysis

DNA synthesis and cell cycle was analyzed using FITC BrdU Flow Kit (Becton, Dickinson and Company, Franklin Lakes, NJ, USA) according to the manufacturer's instructions. In brief, CMT93 cells were seeded onto 6cm dishes with 1.0×10^3^ cells and cultured overnight. Cells were stimulated with or without glucagon for 72 h at 37°C. After stimulation with glucagon, cells were incubated with 10 μM BrdU in cell culture medium for 40 min at 37°C, detached from culture dish, and washed with PBS. Cells were fixed and permeabilized by resuspension in 100 μL BD Cytofix/Cytoperm Buffer. After incubation for 20 min at room temperature, the cells were washed with BD Perm/Wash Buffer, and then incubated in 100 μL BD Cytoperm Plus Buffer for 10 min on ice, followed by another wash. The cells were re-fixed for 5 min at room temperature, washed once, and resuspend in 100 μL of diluted DNase to expose incorporated BrdU for 1 hour at 37°C. After washing, the cells were resuspended and incubated with fluorescent anti-BrdU for 20 min at room temperature, followed by a wash. After staining with 7-AAD, the cells were suspended in 1 mL staining buffer for flow cytometry analysis (Becton-Dickinson FACSCanto).

## SUPPLEMENTARY MATERIALS FIGURES



## Abbreviations

GCGR: glucagon receptor
BrdU: bromodeoxyuridine
RT-PCR: real-time polymerase chain reaction
MAPK: mitogen-activated protein kinase
ERK: extracellular signal-regulated kinase
GLP-1: glucagon-like-peptide-1
AICAR: 5-aminoimidazole-4-carboxamide 1-β-D-ribofuranoside
AMPK: an activator of AMP-activated protein kinase
EMEM: Eagle's minimal essential medium
α-TC1-6: alpha-TC1-6
shRNA: short hairpin RNA
siRNA: small interfering RNA
C57BL/6-DIO: C57BL/6-diet induced obesity
DAB: 3,3′-diaminobenzidine tetrachloride.
