# Supplementary material for: Glucagon promotes colon cancer cell growth via regulating AMPK and MAPK pathways
Source: Oncotarget. 2018 Jan 31;9(12):10650–64. doi: 10.18632/oncotarget.24367 (PMC5828215; doi:10.18632/oncotarget.24367)
Supplement: Supplementary file 1 [file oncotarget-09-10650-s001.pdf]

Glucagon promotes colon cancer cell growth via regulating AMPK and MAPK pathways

MATERIALS AND METHODS

DNA Synthesis and cell cycle analysis

DNA synthesis and cell cycle was analyzed using FITC BrdU Flow Kit (Becton, Dickinson and Company, Franklin Lakes, NJ, USA) according to the manufacturer's instructions. In brief, CMT93 cells were seeded onto 6cm dishes with 1.0×10^3 cells and cultured overnight. Cells were stimulated with or without glucagon for 72 h at 37°C. After stimulation with glucagon, cells were incubated with 10 µM BrdU in cell culture medium for 40 min at 37°C, detached from culture dish, and washed with PBS. Cells were fixed and permeabilized by resuspension in 100 µL BD Cytofix/Cytoperm Buffer. After incubation for 20 min at room temperature, the cells were washed with BD Perm/Wash Buffer, and then incubated in 100 µL BD Cytoperm Plus Buffer for 10 min on ice, followed by another wash. The cells were re-fixed for 5 min at room temperature, washed once, and resuspend in 100 µL of diluted DNase to expose incorporated BrdU for 1 hour at 37°C. After washing, the cells were resuspended and incubated with fluorescent anti-BrdU for 20 min at room temperature, followed by a wash. After staining with 7-AAD, the cells were suspended in 1 mL staining buffer for flow cytometry analysis (Becton-Dickinson FACSCanto).

SUPPLEMENTARY MATERIALS

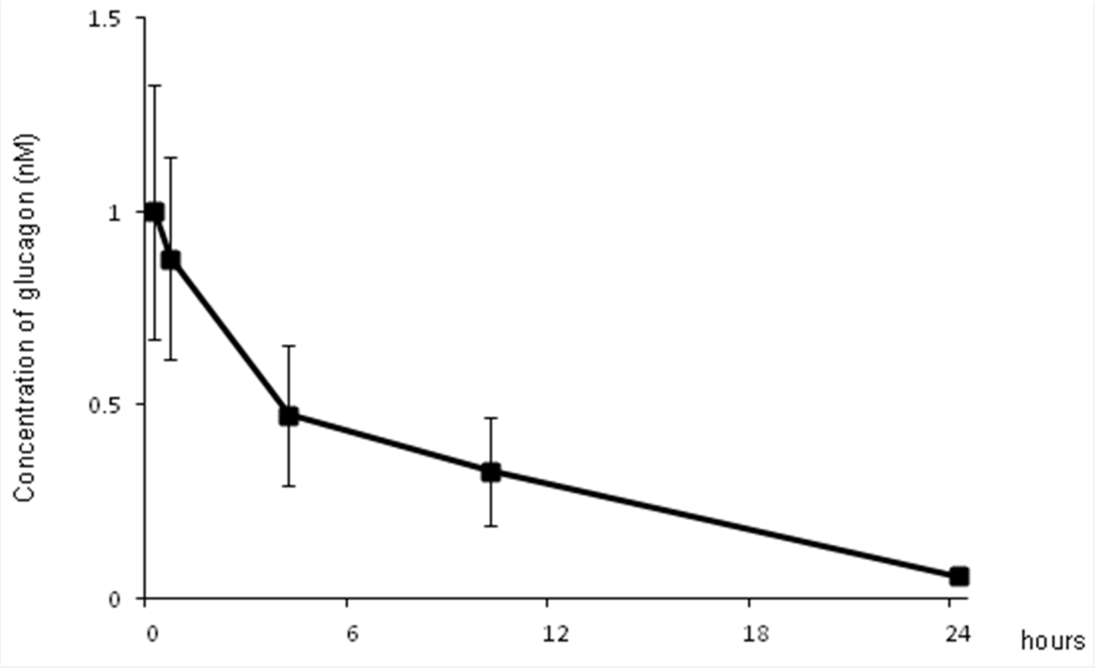

Supplementary Figure 1: The concentrations of glucagon in culture media using for *in vitro* studies were measured by immunoassay using ELISA kit (Mercodia) after the indicated time points after adding 1.0 nM of glucagon into culture media.

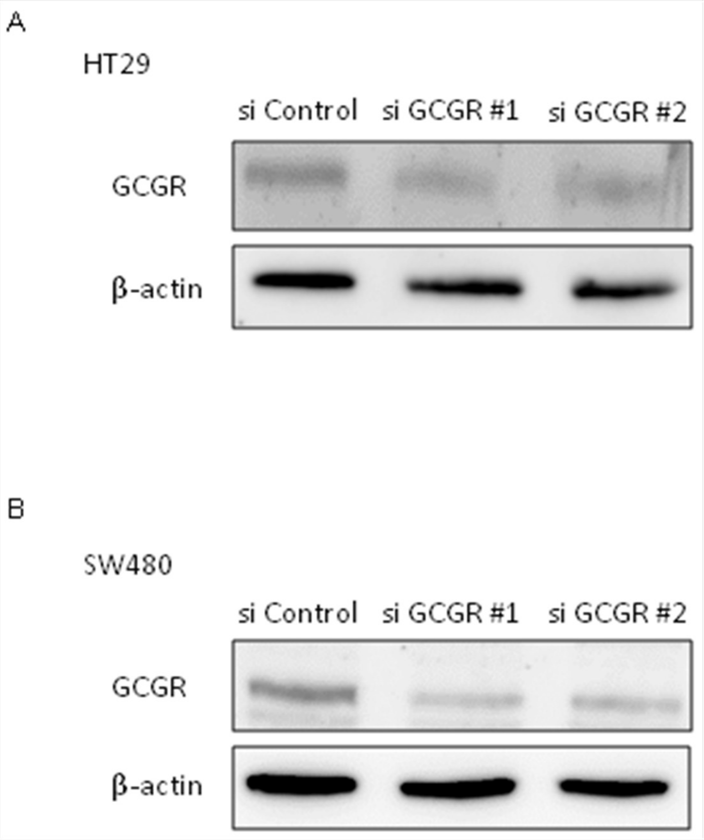

Supplementary Figure 2: Protein extracted from HT29 **(A)** and SW480 **(B)** transfected with siRNA for control or GCGR were immunoblotted with anti-GCGR antibody (Santa Cruz Biotechnology). β-actin (Abcam) was show as a loading control.

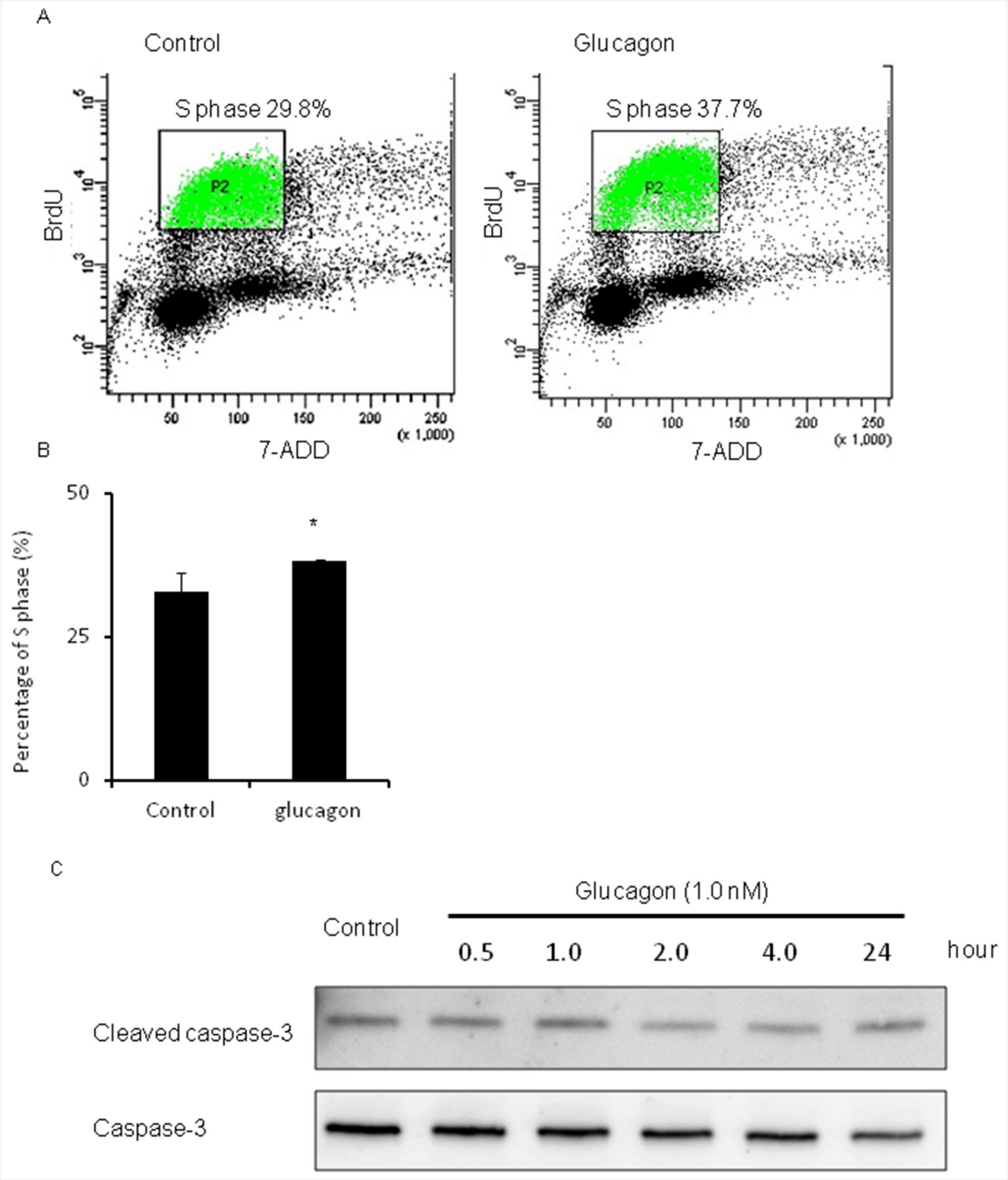

Supplementary Figure 3: (A, B) DNA synthesis and cell cycle assay was performed by BrdU incorporation with 7-AAD staining followed by flow cytometry using CMT93 cells with or without 1.0 nM glucagon stimulation for 72 h. **(C)** Western blot analysis was performed using CMT93 cells treated with 1.0 nM glucagon for 0.5, 1.0, 2.0, 4.0, and 24 hr. Cell lysis were immunoblotted with anti-Cleaved caspase-3 (Asp175) and Caspase-3 antibodies (Cell Signaling Technology). β-actin (Abcam) was show as a loading control. A significant difference is indicated as *($p < 0.05$, Student's *t*-test)

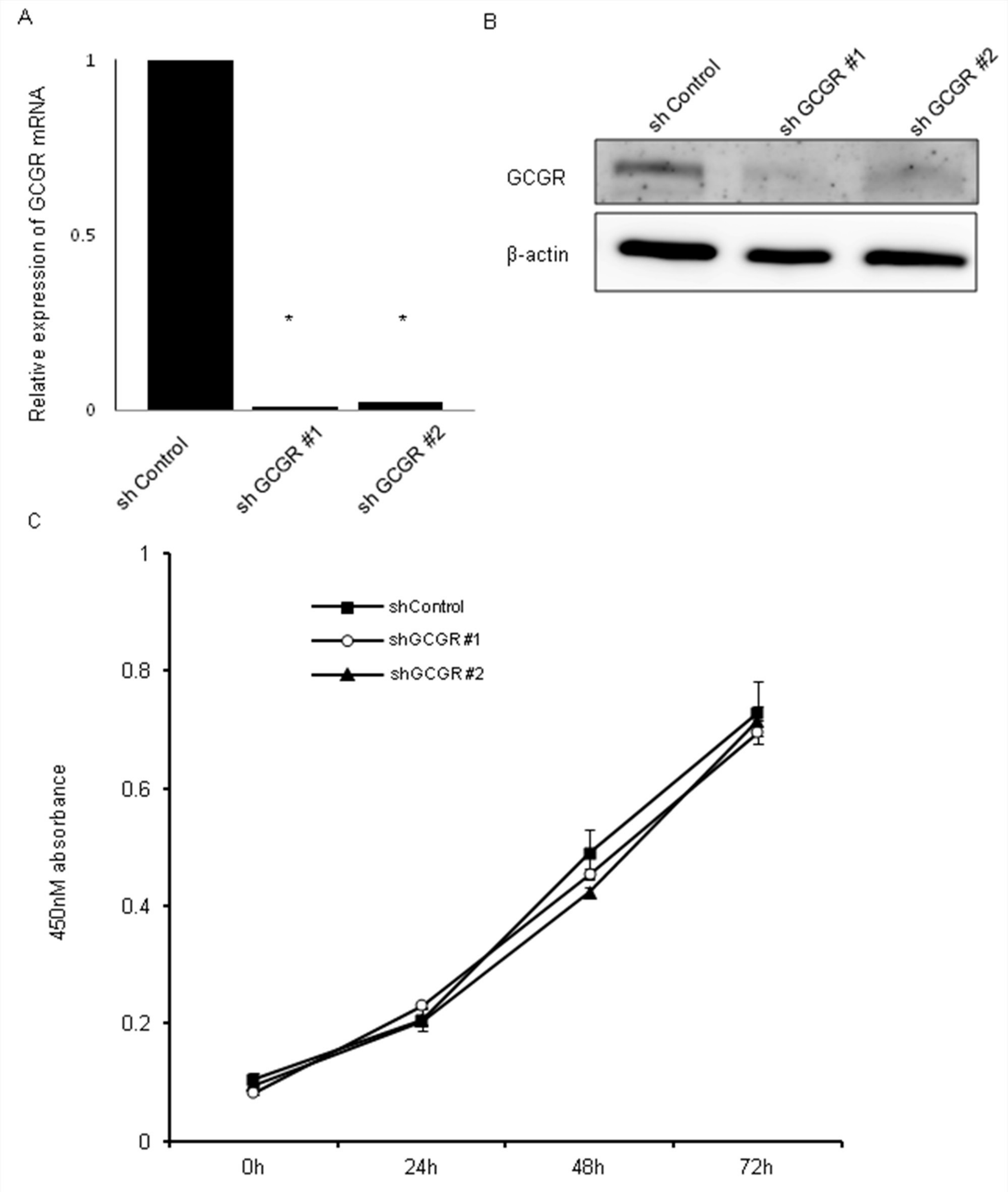

Supplementary Figure 4: (A) RT-PCR was performed using a set of primers targeting GCGR. 34B4 expression was used for normalization. Data are presented as means ± SD, *n*=3. A significant difference is indicated as *($p < 0.05$, ANOVA). **(B)** Protein extracted from CMT93 cells transfected with shRNA for control or GCGR were immunoblotted with anti-GCGR antibody (Santa Cruz Biotechnology). β-actin (Abcam) was show as a loading control. **(C)** 1.0×10^3 CMT93 cells transfected shControl or shGCGR were seeded in 96-well plates. After 24, 48, 72 hours, cell viability was measured using MTT assay.

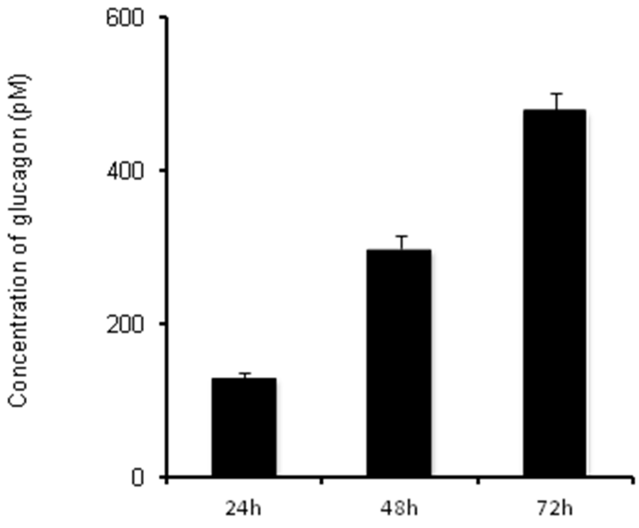

Supplementary Figure 5: The concentrations of glucagon in co-culture media of mouse colon cancer cell line, CMT93 and mouse α cell line, α-TC 1-6 cell were measured by immunoassay using ELISA kit (Mercodia) at the indicated time points after seeding 1.0×10^4 α-TC 1-6 in 24-well plates.

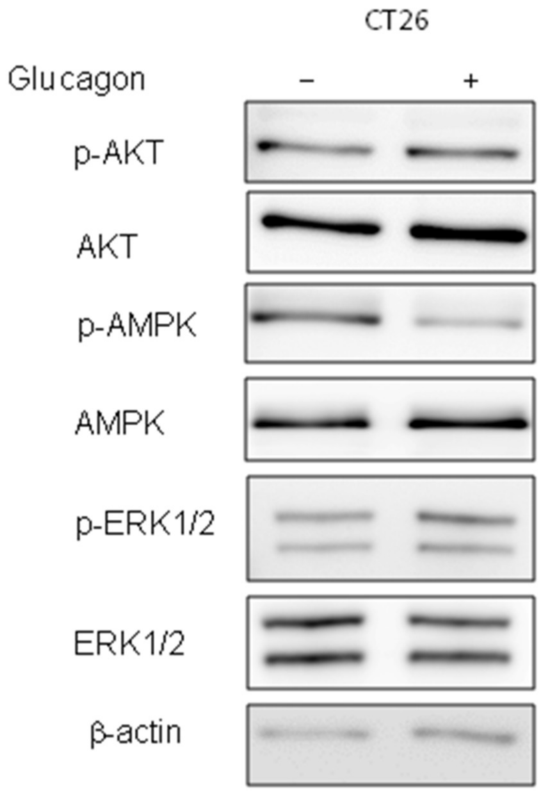

Supplementary Figure 6: Western blot analysis was performed using CT26 cells treated with or without 1.0 nM glucagon for 30 minutes. Cell lysis were immunoblotted with anti-phospho-AKT (Ser473), anti-AKT, anti-phospho-AMPKα (Tyr172), anti-AMPKα, anti-phospho-ERK1/2 (Thr202/Tyr204), anti-ERK1/2 antibodies (Cell Signaling Technology). β-actin (Abcam) was show as a loading control.

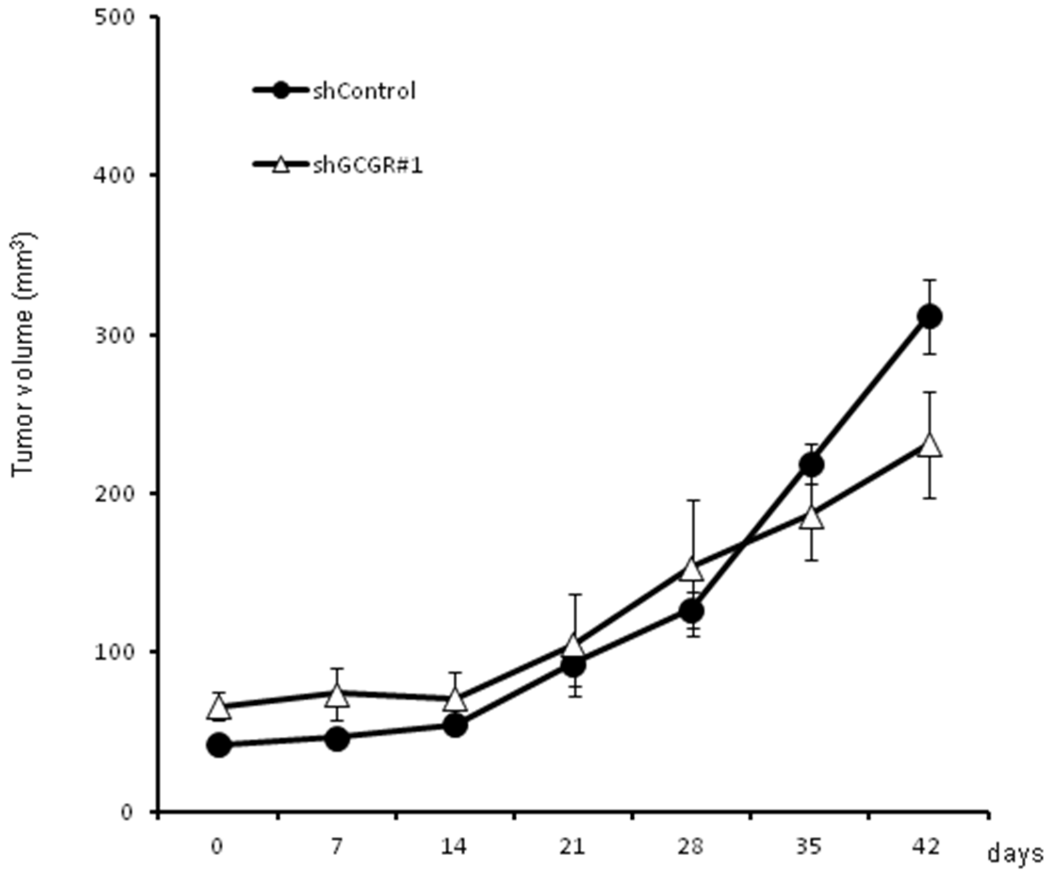

Supplementary Figure 7: 5.0×10^{6} CMT93, control cells, and GCGR knockdown clones were subcutaneously injected into C57BL/6 mice. Tumor growth was measured once a week. Data are presented as means ± SE, 5 animals per group.

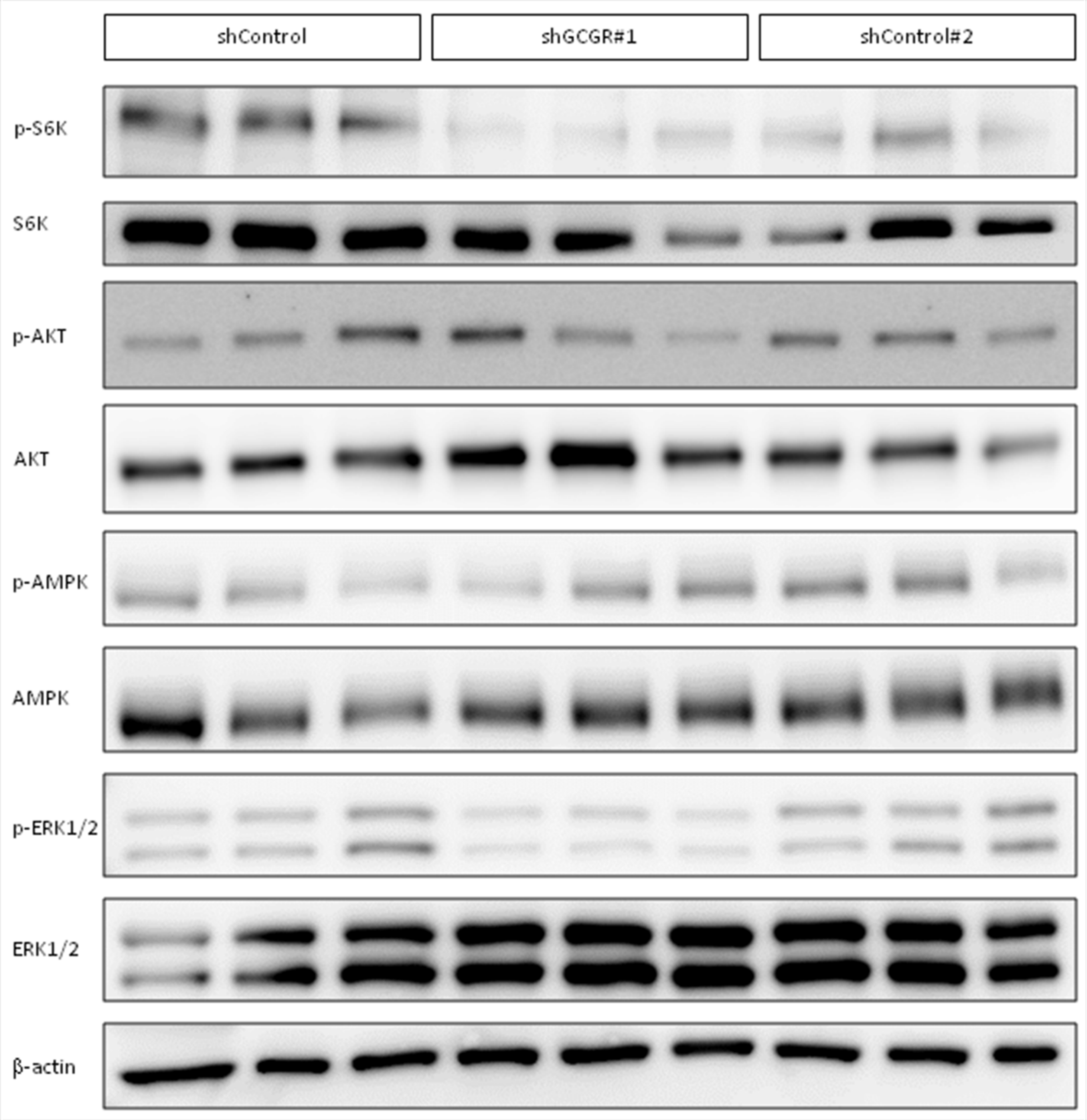

Supplementary Figure 8: Western blot analysis was performed using tumor extracted from mouse allograft model with T2D. Cell lysis were immunoblotted with anti-phospho-S6K (Thr389), anti-S6K, anti-phospho-AMPKα (Tyr172), anti-AMPKα, anti-phospho-ERK1/2 (Thr202/Tyr204), anti-ERK1/2 antibodies (Cell Signaling Technology). β-actin (Abcam) was show as a loading control.

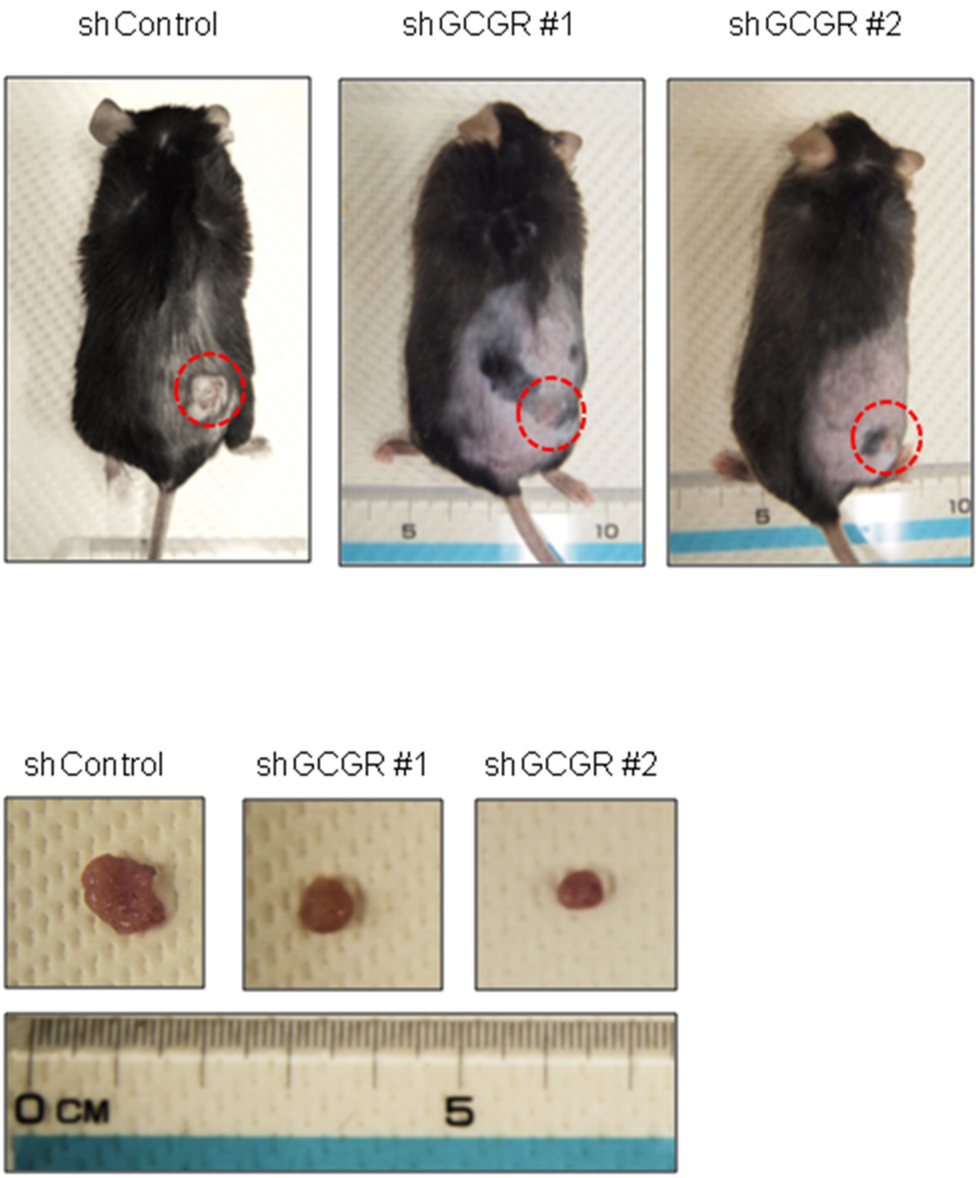

Supplementary Figure 9: 5.0×10[6] CMT93, control cells, and GCGR knockdown clones were subcutaneously injected into C57BL/6-DOI mice at the age of 10 weeks. After euthanasia, transplanted tumors are extracted from mice at the age of 17 weeks.
